# Supplementary material for: Emissive Pentacene-Loaded βcyclodextrin-Derived C-Nanodots Exhibit Red-Light Triggered Photothermal Effect
Source: Pharmaceutics. 2025 Apr 22;17(5):543. doi: 10.3390/pharmaceutics17050543 (PMC12114991; doi:10.3390/pharmaceutics17050543)
Supplement: Supplementary file 1 [file pharmaceutics-17-00543-s001.zip › pharmaceutics-3550410-supplementary.pdf]

# Supporting information

**Ludovica Maugeri [1,†], Giorgia Fangano [1], Ester Butera [1,†], Giuseppe Forte [1], Paolo Giuseppe Bonacci [2], Nicolò Musso [3], Francesco Ruffino [4], Loredana Ferreri [5], Grazia Maria Letizia Consoli [5] and Salvatore Petralia [1,5,6,7,*]**

1 Department of Drug and Health Sciences, University of Catania, Via Santa Sofia 64, 95125 Catania, Italy
2 Department of Biomedicals and Biotechnologies Sciences, University of Catania, Via Santa Sofia 89, 95123 Catania, Italy
3 Faculty of Medicine and Surgery, "Kore" University of Enna, Contrada Santa Panasia, 94100 Enna, Italy
4 Department of Physic and Astronomy, University of Catania, Via Santa Sofia 64, 95125 Catania, Italy
5 CNR-Institute of Biomolecular Chemistry, Via Paolo Gaifami 18, 95126 Catania, Italy
6 NANOMED, Research Centre for Nanomedicine and Pharmaceutical Nanotechnology, University of Catania, Viale A. Doria 6, 95124 Catania, Italy
7 CIB-Interuniversity Consortium for Biotechnologies U.O. of Catania, Via Flavia, 23/1, 34148 Trieste, Italy
* Correspondence: salvatore.petralia@unict.it
† These authors contributed equally to this work.

---

## Content:

**Figure S1.** Different reaction times (45 min, 1.5 h, and 3.0 h) of CDs-βCD synthesis.
**Figure S2.** AFM full scan (0.3 × 0.3 μM) image and cross section profile for Cdots-βCD.

**Photothermal conversion efficiency (η)**

**Figure S3.** Linear relationship between time (sec) and -ln($\theta$) for CDs-βCD.
**Figure S4.** Emission spectrum at excitation wavelength at 400 nm for CDs-βCD solution (black) and for CDs-βCD/PTC dispersion (red).
**Figure S5.** AFM full scan (0.3 × 0.3 μM) image and cross section profile for Cdots- βCD/PTC.
**Figure S6.** Linear relationship between time (sec) and -ln($\theta$), the slope is the time constant ($\tau$s) for CDs-βCD/PTC.

**Computational Methods**

**Figure S7.** Initial complexes geometries C1, C2 and C3.
**Figure S8.** Final geometries, referred to as, C1, C2 and C3, from left to right at B3LYP/6-311+G(d,p) /CPCM level of theory
**Table S1**. Free energy of formation, ΔGf, (kcal mol-1) for the studied complexes; adsorption $\lambda$ (nm), oscillator strength, $f$, and transition related to UV-vis spectra of C1.
**Table S2.** Percentage of growth compared to control (CTRL) of cells treated with decreasing amounts of CDs-βCD/PTC.
**Table S3.** Percentage of growth compared to control (CTRL) of cells treated with decreasing amounts of Cdots-βCD/PTC.
**Figure S9.** DLS measurements of the Cdots-βCD nanostructures
**Figure S10.** DLS measurements of the Cdots-βCD/PTC nanostructures
**Figure S11.** Emission spectrum (excitation wavelength at 680 nm) for Cdots-βCD solution (red line) and for Cdots-βCD/PTC dispersion (blu line).

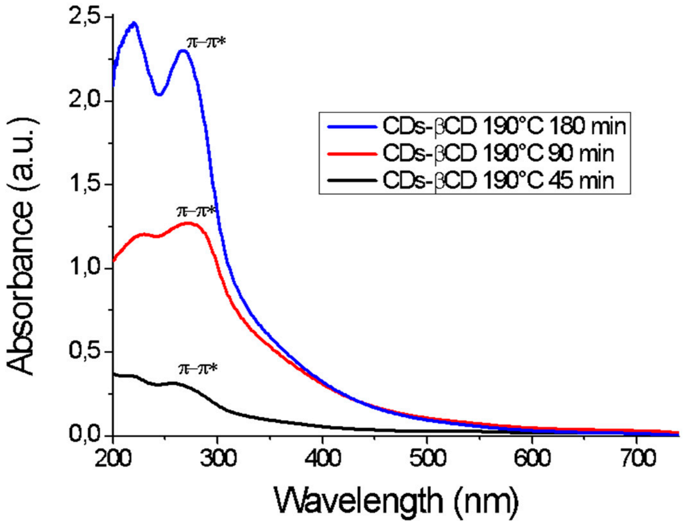


**Figure S1.** Different reaction times (45 min, 1.5 h, and 3.0 h) of CD-βCD synthesis.

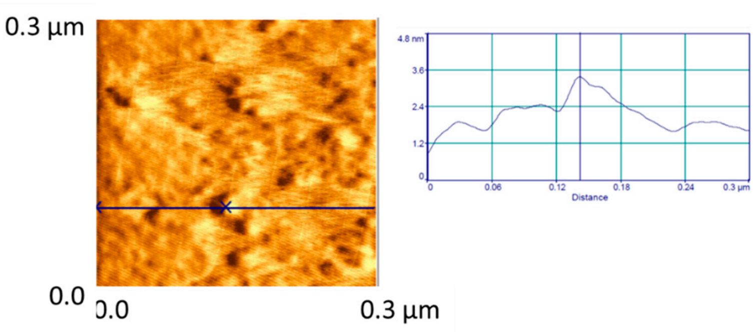


**Figure S2** AFM full scan (0.3 × 0.3 µM) image and cross section profile for Cdots- βCD revealed the presence of spherical Carbon core with size in the range 2.5 ± 0.2 nm, in large aggregates.

**Photothermal Conversion Efficiency (η).**

The photothermal conversion efficiency (η) was calculated according to equation (1) introduced by Roper:

$$\eta = \frac{hA\,(T_{max} - T_{surr}) - Q_{Dis}}{I(1 - 10^{-A})} \quad \text{(S1)}$$

where Tmax (29.4 °C) and Tsurr (22.6 °C) represents the max photothermal temperature and the ambient temperature respectively, I is the incident laser power. The absorbance (A=0.56) of Cdots-βCD at 405 nm. The equations (2) and (3) were introduced to calculate the parameter hA.

$$\theta = \frac{T - T_{surr}}{T_{max} - T_{surr}} \tag{S2}$$

$$\tau = \frac{M_D \, C_D}{hA} \tag{S3}$$

where MD and CD are the mass (0.1 g) and heat capacity (4.2 J $g^{-1}$) of water respectively, and $\tau$s is the time constant, calculated by the equation (4).

$$t = -\tau_s(ln\theta) \tag{S4}$$

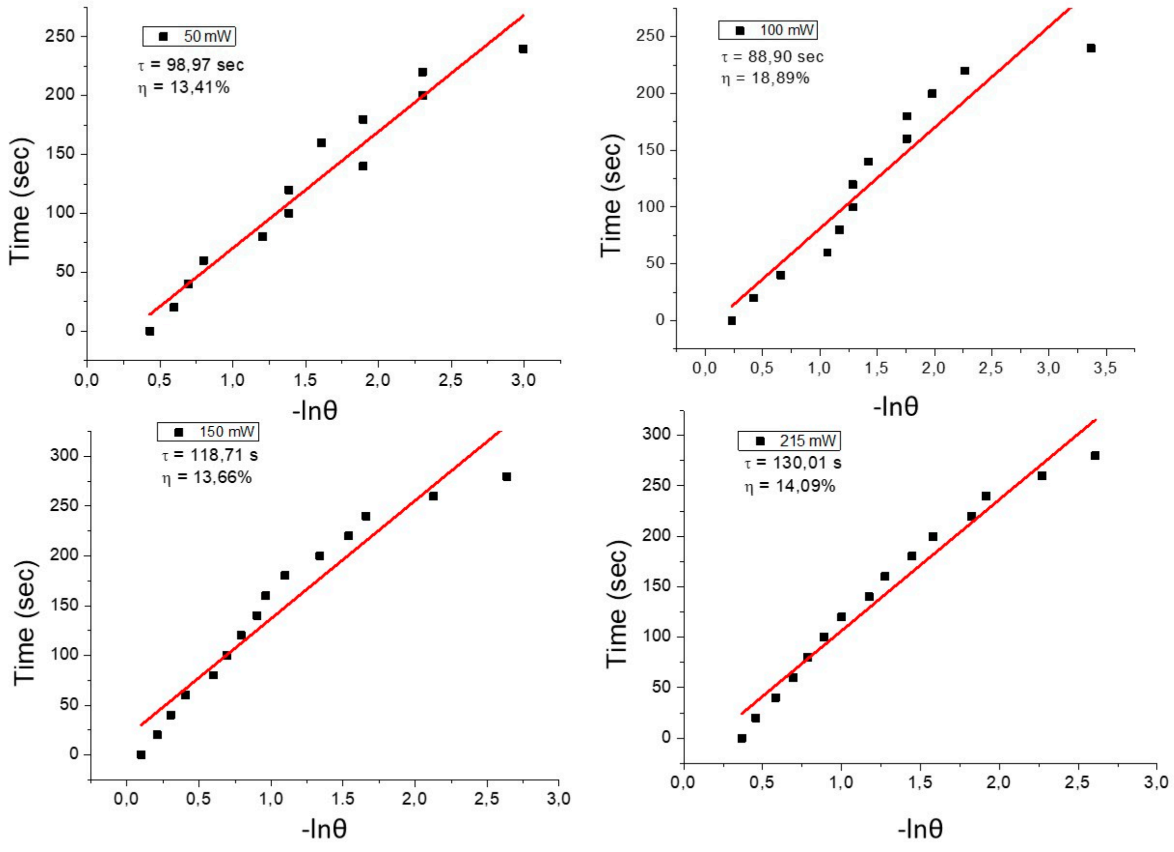


**Figure S3.** Linear relationship between time (sec) and -ln$\theta$, the slope is the time constant ($\tau_s$) for Cdots-βCD.

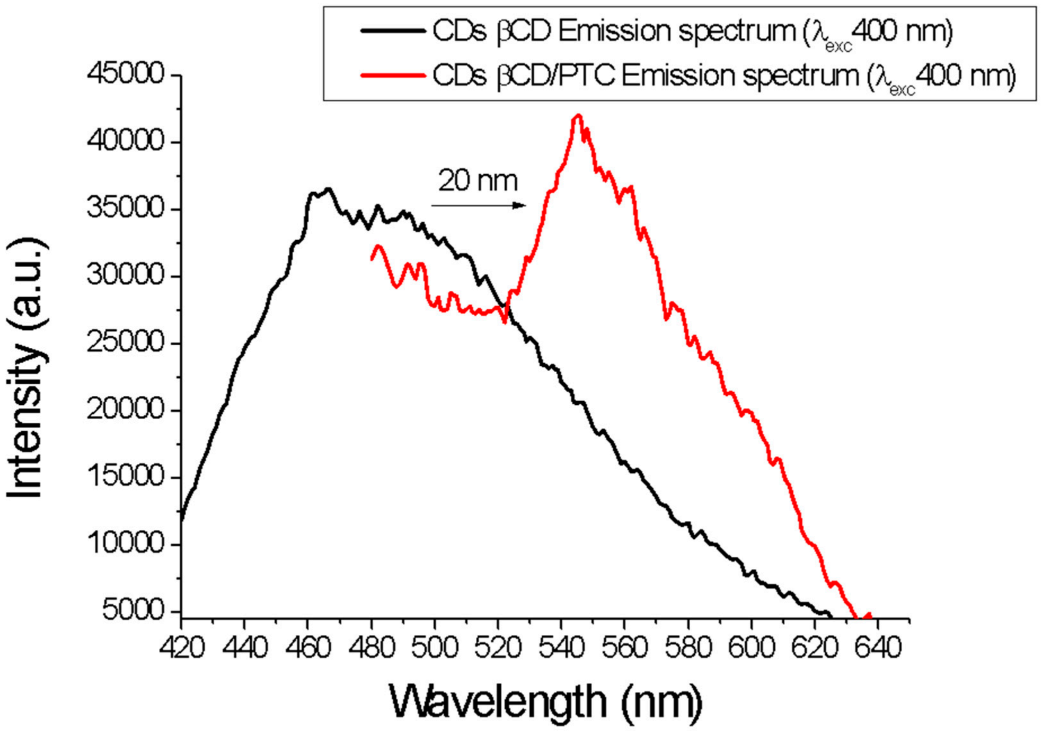


**Figure S4.** Emission spectrum at excitation wavelength at 400 nm for Cdots-βCD solution (black) and for Cdots-βCD/PTC dispersion (red).

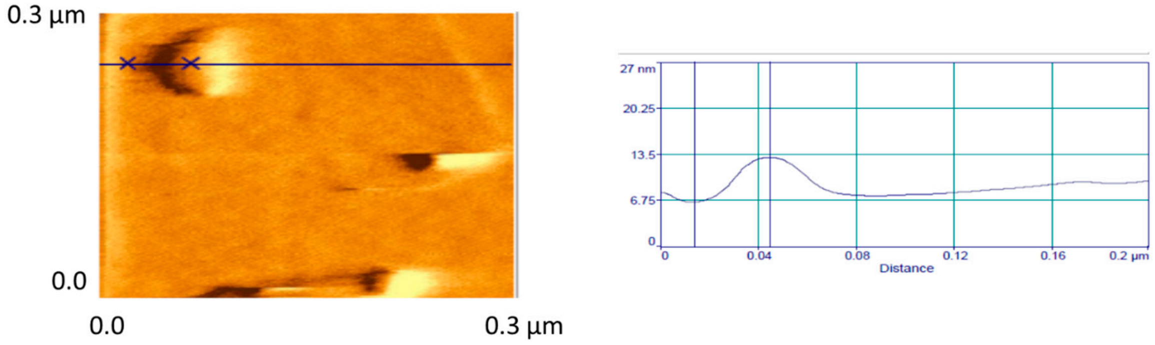


**Figure S5**. AFM full scan (0.3 × 0.3 µM) image and cross section profile for Cdots-βCD/PTC revealed the presence of spherical nanostructures with size in the range 8.2 ± 0.8 nm.

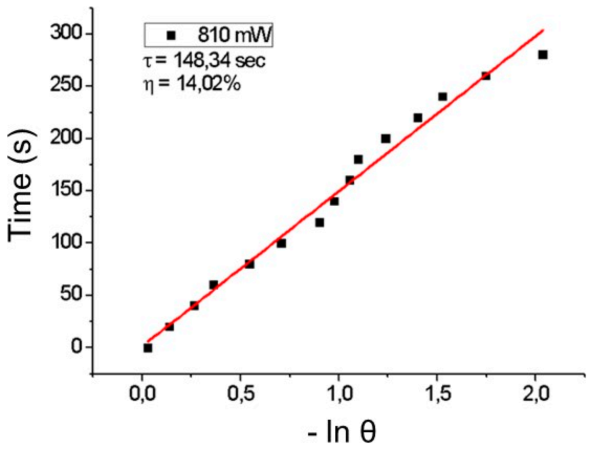


**Figure S6.** Linear relationship between time (sec) and -ln($\theta$), the slope is the time constant ($\tau_s$) for Cdots-βCD/PTC.

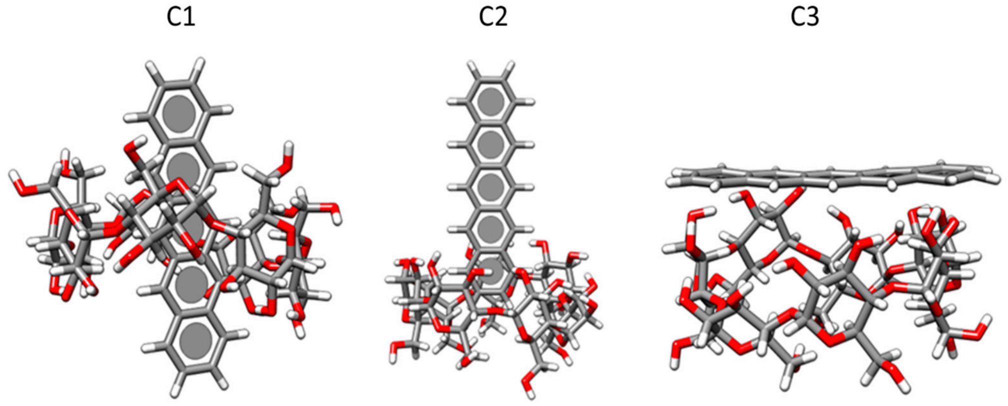


**Figure S7.** Initial complexes geometries C1, C2 and C3.

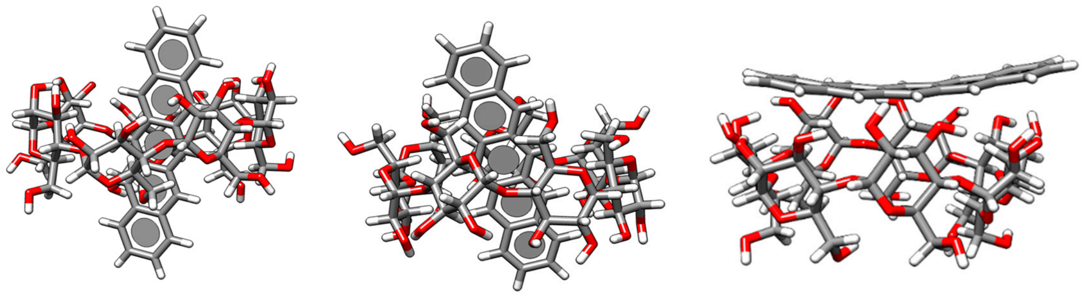

**Figure S8.** Final geometries, referred to as, C1, C2 and C3, from left to right at B3LYP/6-311+G(d,p) /CPCM level of theory.

**Table S1:** Free energy of formation, **ΔGf**, (kcal mol$^{-1}$) for the studied complexes; adsorption $\lambda$ (nm), oscillator strength, $f$, and transition related to UV-vis spectra of C1

| Complex | ΔGf | λ | f | Transition |
|---|---|---|---|---|
| **C1** | -9.35 | 683.51 | 0.21 | S0->S1 |
| | | 291.23 | 2.59 | S0->S4 |
| **C2** | -9.71 | | | |
| **C3** | -5.32 | | | |

**Table S2:** Percentage of growth compared to control (CTRL) of cells treated with decreasing amounts of Cdots-βCD.

| Two-stage linear step-up procedure of Benjamini, Krieger and Yekutieli | Mean Diff, | Discovery? | q value | Individual *p* Value |
|---|---|---|---|---|
| CTRL vs. 0.8μg | -0.02325 | No | 0.5055 | 0.2848 |
| CTRL vs. 0.08μg | 0.004750 | No | 0.8641 | 0.8229 |
| CTRL vs. 0.008μg | 0.02150 | No | 0.5055 | 0.3209 |

**Table S3:** Percentage of growth compared to control (CTRL) of cells treated with decreasing amounts of Cdots-βCD/PTC.

| Two-stage linear step-up procedure of Benjamini, Krieger and Yekutieli | Mean Diff, | Discovery? | q value | Individual *p* Value |
|---|---|---|---|---|
| CTRL vs. 0.8μg | 0.2022 | Yes | 0.0003 | 0.0001 |
| CTRL vs. 0.08μg | 0.05620 | No | 0.1970 | 0.1876 |
| CTRL vs. 0.008μg | 0.01380 | No | 0.5178 | 0.7398 |

**Figure S9** DLS measurements of the Cdots-βCD nanostructures

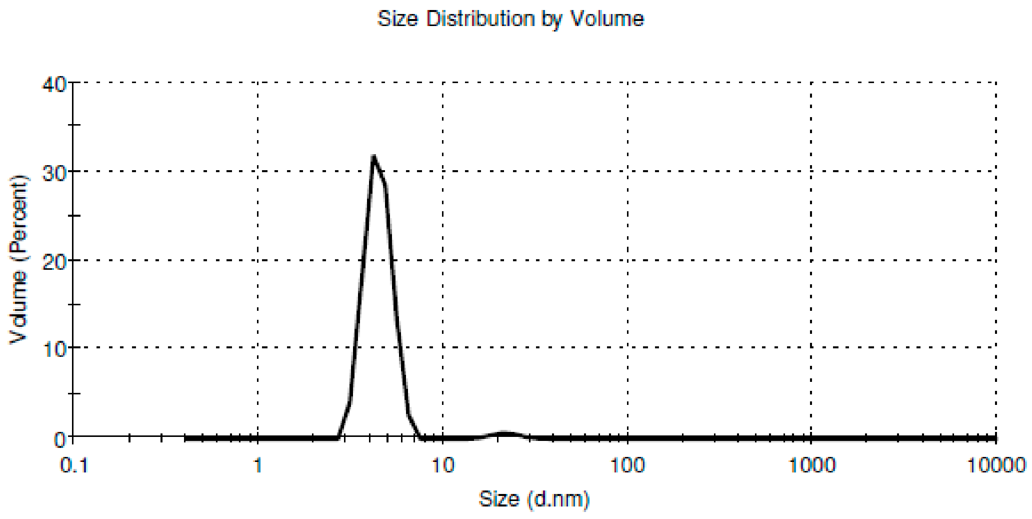


**Figure S10** DLS measurements of the Cdots-βCD/PTC nanostructures

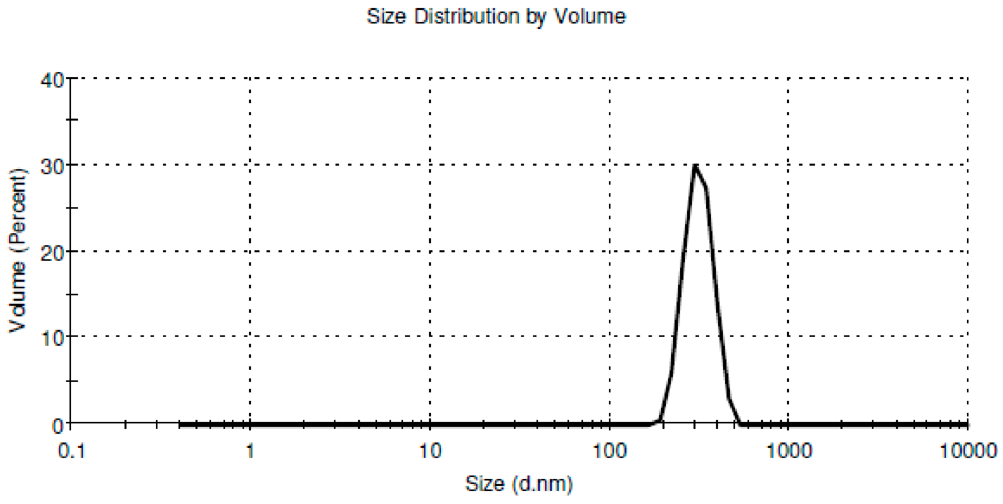

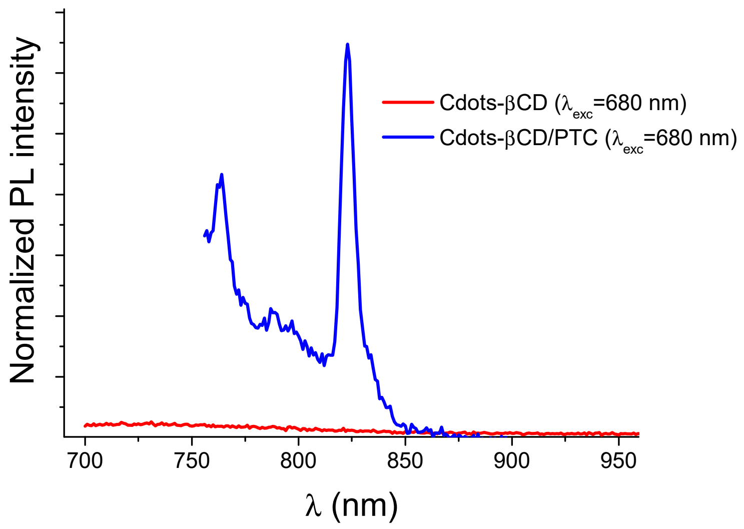


**Figure S11.** Emission spectrum (excitation wavelength at 680 nm) for Cdots-βCD solution (red line) and for Cdots-βCD/PTC dispersion (blu line).
